# Adenocarcinoma of the Right Breast in a Man with Intellectual Disability

**DOI:** 10.1155/2013/968035

**Published:** 2013-07-11

**Authors:** Daniel Satgé, Bernard Leduc, Fernand Raffi, Etienne Roux

**Affiliations:** ^1^ONCODEFI Project Epidemiology and Biostatistics Department (EA 2415), Laboratoire Biostatistique Epidémiologie Santé Publique (EA 2415), University Institute for Clinical Research IURC, Montpellier 1 University, 34093 Montpellier Cedex 5, France; ^2^Medical Oncology, Centre Hospitalier, 19100 Brive-La-Gaillarde, France; ^3^Gynecology-Obstetrics, Centre Hospitalier, 19000 Tulle, France

## Abstract

A 64-year-old man with moderate intellectual disability developed a large right breast carcinoma with lymph node metastases. Cancer treatment is often difficult in persons with intellectual disability. However, the patient could be treated according to the current protocols with surgery, chemotherapy, and radiotherapy. He is alive and in good health two years after discovery of his tumor. Although breast cancer is estimated as frequent in women with intellectual disability as it is in nondisabled women, our patient is the second man with intellectual disability reported with a breast carcinoma.

## 1. Introduction

Breast cancer risk in women with intellectual disability (ID) is currently estimated to be the same as in women in the general population [1, 2]. Male breast cancer is nearly 100 times less frequent than breast cancer in women in the general population [3, 4]. To our knowledge, only one man with intellectual disability (MWID) and breast cancer has been reported [5]. We present a new case and underline the need to keep this possible association in mind.

## 2. Case Report

**Figure 1:**
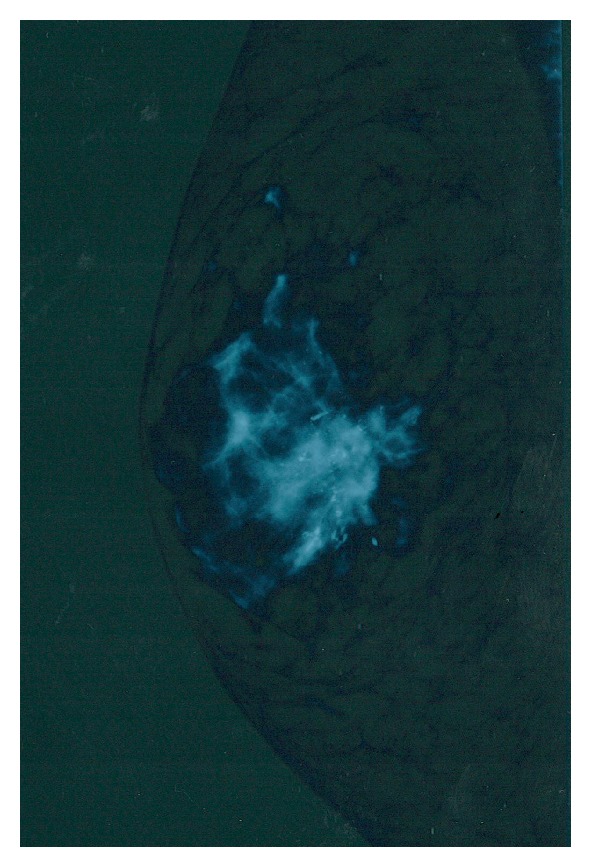
Mammography of the right breast occupied with a large carcinoma.

A 64-year-old 83 kg and 1.74 m man was referred for a lump that he discovered himself in his right breast. He lived in an institution due to a moderate ID which appeared following seizures in childhood. He is not dysmorphic, and his karyotype is normal. No genetic (FISH, CGH) or particular neuroimaging studies have been conducted. His maternal grandfather died from throat cancer. One of his three sisters died from liver cancer linked to alcohol abuse. The two other sisters and his younger brother are in good health. There is no family history of breast cancer. The patient has no personal history of testicular or liver disease. He is treated for knee osteoarthritis. He is regularly followed for raised PSA serum levels at 5.37 ng/mL, but biopsies were negative for prostate carcinoma. On physical examination the patient was in good general health. A 4 × 3 cm mobile indurated mass was palpated on his right breast without nipple discharge or skin modification. Enlarged right axillary lymph nodes were palpated. The left breast was normal. Mammography showed a breast carcinoma (Figure 1). A core needle biopsy revealed a poorly differentiated grade 3 invasive ductal carcinoma. Preoperative checkup did not find metastases. The 15 × 13 × 4 cm right mastectomy contained a 5.3 × 4.4 cm invasive ductal carcinoma with foci of intraductal carcinoma and aspects of perineural invasion. Tumor cells were found in small lymphatic vessels. Surgical margins were free of neoplastic tissue. Estrogen and progesterone receptors were positive; C-erbB-2 was overexpressed (score 3 on 3). The axillary dissection contained 28 lymph nodes, 18 of which were metastasized with capsule rupture. The patient's disease and treatment were explained to him and he agreed for the therapy. For his T_2_ N_2 _M_0_ disease the patient received postmastectomy radiotherapy on the surgical field and on local lymphatic areas. He also received 4 courses of docetaxel and cyclophosphamide associated with trastuzumab, followed by 4 courses epirubicin, 5FU, and cyclophosphamide. Despite his ID the patient could receive the usual treatment. He is now in good health two years after the discovery of his tumor.

## 3. Discussion

We could find only one published observation of breast cancer in an MWID. A 66-year-old man with hypothyroidism-linked severe mental retardation and thyroid carcinoma had a 2.5 cm infiltrating ductal carcinoma in his left breast and a 1.5 markedly atypical papillary lesion in his right breast [5]. The true frequency of breast carcinoma in MWID remains unknown. If observations of breast cancer in MWID are not reported in the medical literature, this may lead to the false impression that the disease is even rarer than in nondisabled men. Some genetic conditions more frequently associated with ID such as Cowden syndrome, Saethre-Chotzen syndrome, Klinefelter syndrome, and type 1 neurofibromatosis [6–9] are at higher risk of breast cancer. Additionally, a genetic microdeletion may be associated with mental retardation and breast cancer in a family [10]. Men with these syndromes could be followed more closely for breast cancer. It is interesting to note that gynecomastia which is sometimes considered as a risk factor for male breast cancer was estimated rarer in MWID, except for men with Down syndrome who had a similar frequency of gynecomastia as that in the general population [11]. In our patient we did not identify a personal risk factor for male breast cancer such as overweight, testicular disorder, or exposure to toxic exogenous factors. We also did not find a predisposing genetic condition or a family history of breast carcinoma. 

Due to psychological reasons linked to difficulties to understand and communicate, cancer treatment is often complicated in persons with ID. Treatment must sometimes be modified, for instance, due to the necessity to respect time of immobility for radiotherapy and chemotherapy [12]. Our observation shows that patients with ID may nonetheless benefit from usual therapeutic protocols. This observation also shows that any man with ID, even without a recognized predisposing condition, may develop a breast cancer. In our patient who discovered himself the tumor, the age at diagnosis and clinical presentation was the same as in the general population. However, the tumor was already 5 cm large at diagnosis. For more deeply intellectually disabled men, the discovery of a breast cancer will rely on professional and familial carers. 

## 4. Conclusion

As breast is a superficial organ and as breast cancer prognosis is closely linked to the tumor stage, an early diagnosis is possible and important. Since treatment strategies for breast cancer are well defined and since the disease can be cured, we should also keep in mind that MWID may present a breast carcinoma.
